# Case report: Bilateral sudden deafness in acute middle cerebellar peduncle infarction: central or peripheral?

**DOI:** 10.3389/fmed.2023.1174512

**Published:** 2023-05-05

**Authors:** Ziyun Yuan, Lei Xiang, Ran Liu, Wei Yue

**Affiliations:** ^1^Clinical College of Neurology, Neurosurgery and Neurorehabilitation, Tianjin Medical University, Tianjin, China; ^2^Department of Neurology, Tianjin Huanhu Hospital, Tianjin, China

**Keywords:** bilateral sudden sensorineural hearing loss, middle cerebellar peduncle infarction, anterior inferior cerebellar artery, audiological examination, cochlear, prognosis

## Abstract

**Background:**

The middle cerebellar peduncle (MCP) is the most common site associated with hearing impairment in acute ischaemic stroke. Narrowing or occlusion of the vertebrobasilar artery due to atherosclerosis is thought to be the main pathogenesis of MCP infarction. Most previous reports of MCP infarction have not been clear whether the patient's hearing impairment is localized to the center or periphery.

**Case presentation:**

We report 44-year-old man with vertigo, tinnitus, and bilateral sudden sensorineural hearing loss (SSNHL) as the first symptoms. Pure Tone Audiogram revealed complete hearing loss in both ears. Acute bilateral MCP infarction was diagnosed by repeated brain magnetic resonance imaging (MRI). The brainstem auditory evoked potential (BAEP) and the electrocochleography were normal. The otoacoustic emissions showed binaural cochlear dysfunctions. After the antiplatelet, lipid-lowering, steroids and hyperbaric oxygen therapy, the pure-tone average (PTA) showed a clear improvement with 67 decibels (dB) on the right and 73 dB on the left at the 3-month follow-up.

**Conclusion:**

Vertebrobasilar diseases due to atherosclerosis should be routinely considered in middle-aged and elderly patients with vascular risk factors and bilateral hearing loss. Bilateral SSNHL can be a prodrome of acute MCP infarction and it can be peripheral. Brain MRI, brain magnetic resonance angiogram (MRA), brain and neck computed tomography angiography (CTA), BAEP, otoacoustic emissions, and Pure Tone Audiogram help to localize and qualify the diagnosis. Bilateral SSNHL localized to the periphery usually improves better and has a good prognosis. Early detection of hearing loss and intervention can help patients recover.

## 1. Introduction

SSNHL refers to a rapid-onset hearing loss occurring within 3 days, with hearing loss ≥30 dB in at least three contiguous audiometric frequencies (1). The global annual incidence of SSNHL is approximately 5–27 per 100,000 people, in which hearing impairment is commonly unilateral (2). Viral infection, vascular occlusion, and autoimmune diseases have been implicated as causes of SSNHL, with vascular occlusion being one of the most common causes in older patients (3). The internal auditory artery (IAA), which supplies blood to the inner ear, originates from the vertebral basilar artery system, commonly from the anterior inferior cerebellar artery (AICA), or less commonly from the basilar artery (BA), and infrequently from the posterior inferior cerebellar artery (PICA). The IAA is a long and slender terminal artery without collateral circulation, which is why sudden deafness is possibly a prodrome of AICA stenosis or occlusion. Almost all the MCP is supplied by the AICA; therefore, bilateral MCP infarcts may be accompanied by hearing loss. Patients with MCP infarction who present with SSNHL are less likely to be reported due to the omission of audiological examination, especially in the case of bilateral hearing loss where patients tend to overlook mild hearing loss. And the hearing loss revealed by PTA alone in some cases is not sufficient to confirm whether it is central or peripheral. Here, we report on a patient with vertigo, tinnitus, and SSNHL as a prodrome of acute MCP infarction, and demonstrate the localization of bilateral deafness.

## 2. Case presentation

**Figure 1:**
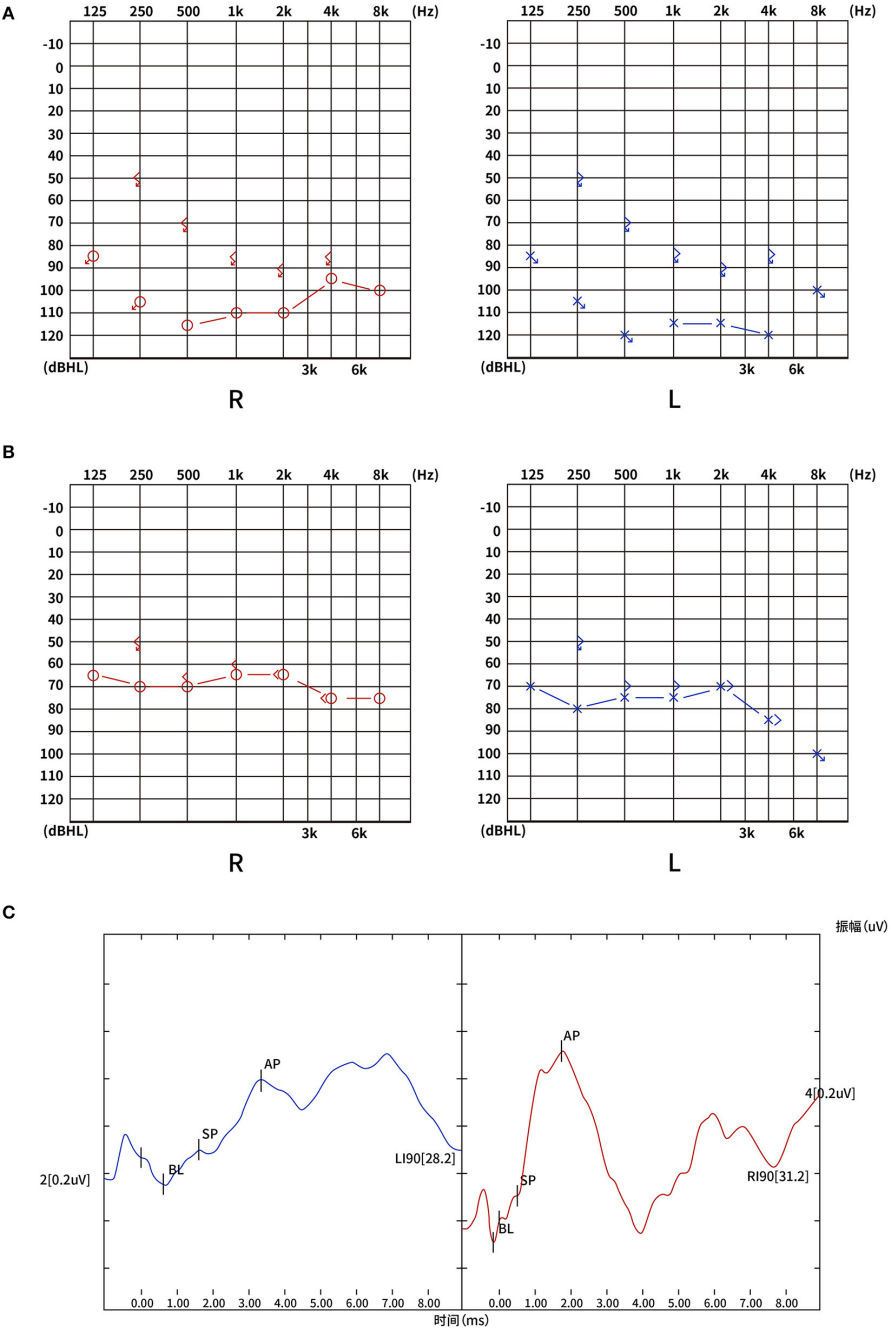
**(A)** PTA on admission: total SSNHL in both ears: 112 dB on the right and 115 on the left. **(B)** PTA after 3-month follow-up: severe hearing loss in both ears: 67 dB on the right and 73 on the left. **(C)** The electrocochleogram showed the binaural SP/AP ratio in the normal range at 90 dBnHL, with the left ear SP/AP ratio of 0.33 and the right ear SP/AP ratio of 0.25. PTA, pure-tone average; SSNHL, sudden sensorineural hearing loss; dB, decibel; SP, summating potential; AP, action potential; dBnHL, decibel normal hearing level; R, right; L, left.

A 44-year-old man with hypertension, suddenly developed vertigo, tinnitus, and bilateral hearing loss 1 day before admission. The patient was hospitalized in Tianjin Huanhu Hospital after the above symptoms persisted without improvement. Up on physical examination, he did not have dysarthria, limb weakness, ataxia, or spontaneous nystagmus, but sustained horizontal gaze-evoked nystagmus to the right or left in both eyes, abnormal horizontal head impulse test and absent skew. PTA showed a total SSNHL of 112 dB in the right ear and 115 dB in the left ear (Figure 1A), and no infarct foci were found on the head MRI.

**Figure 2:**
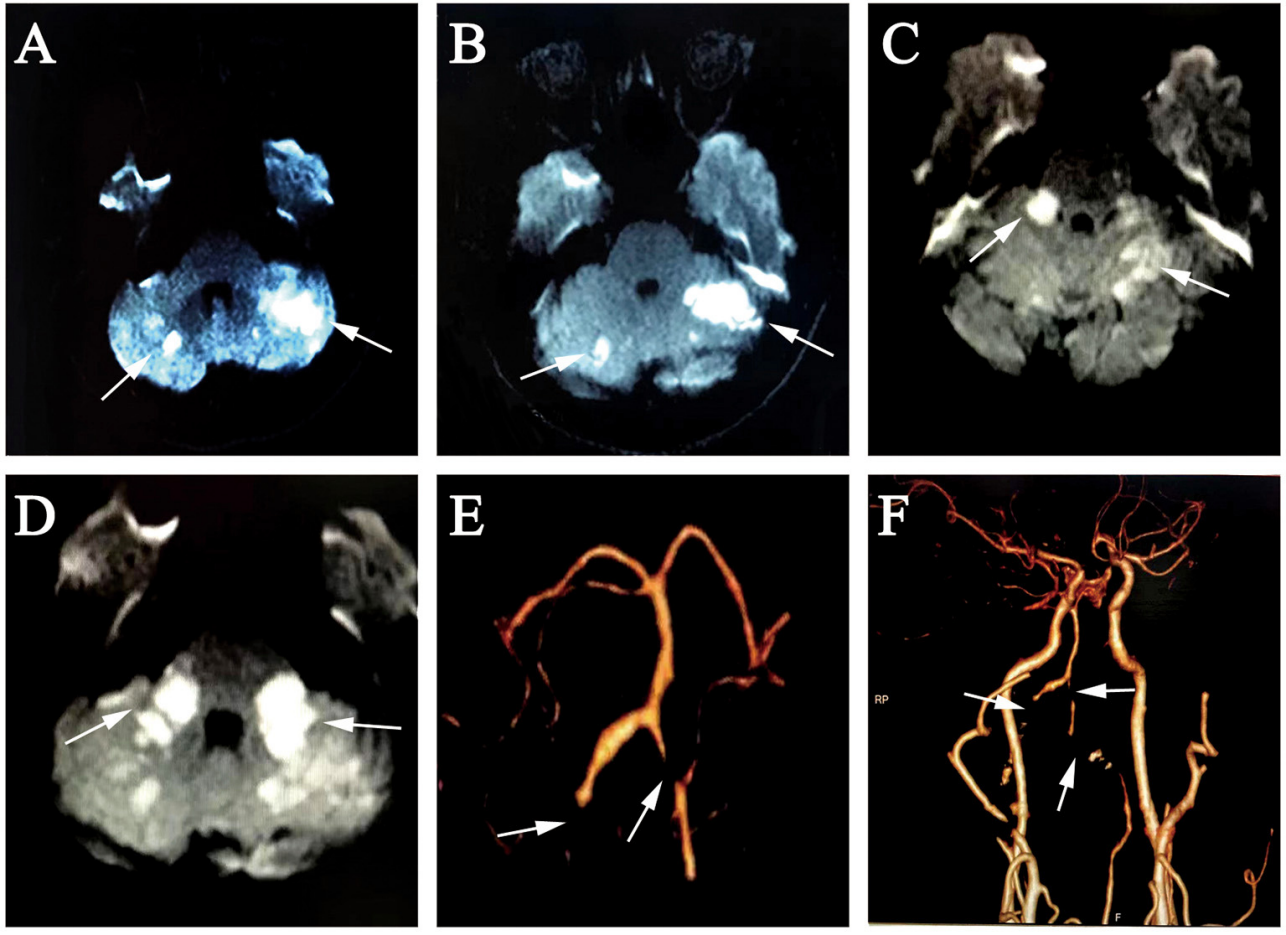
**(A, B)** 3rd day, diffusion-weighted MRI showed hyperintense foci at the bilateral cerebellum; **(C, D)** 10 days later, diffusion-weighted MRI showed new hyperintense foci at the bilateral MCP; **(E, F)** brain and neck CTA revealed occlusion of the bilateral VA V3 segment, proximal V4 segment and bilateral AICA, severe stenosis of the distal left VA V4 segment. MRI, magnetic resonance imaging; MCP, middle cerebellar peduncle; CTA, computed tomography angiography; VA, vertebral artery; AICA, anterior inferior cerebellar artery.

Three days after the initial onset of the hearing loss and vertigo, the patient's symptoms worsened. Clinical neurological examination revealed cerebellar dysarthria, gaze-evoked nystagmus, and ataxia. Reexamination of the brain MRI demonstrated an acute infarction in the bilateral cerebellum (Figures 2A, B).

After 10 days of hospitalization, the patient developed left-limb weakness and worsening ataxia. A brain MRI revealed a new acute infarction extending to the bilateral MCP (Figures 2C, D). Laboratory results indicated hyperlipidemia with plasma total cholesterol (TC) 7.05 mmol/L and low-density lipoprotein cholesterol (LDL-C) 4.30 mmol/L. Brain and neck CTA showed occlusion of the bilateral vertebral artery (VA) V3 segment, proximal V4 segment, and bilateral AICA as well as severe stenosis of the distal left VA V4 segment (Figures 2E, F). The BEAP showed normal elicitation of I, III, and V waves, as well as normal waveforms and amplitudes in each wave, and latencies and interpeak latencies within the normal range on day 14. The electrocochleogram showed the binaural SP/AP ratio in the normal range at 90 dBnHL, with the left ear SP/AP ratio of 0.33 and the right ear SP/AP ratio of 0.25 (Figure 1C). The tympanogram type was A, without the stapedial reflex bilaterally. Transient evoked otoacoustic emissions and distortion product otoacoustic emissions showed binaural cochlear dysfunctions. Bithermal caloric test indicated bilateral horizontal semicircular canal low-frequency dysfunction.

**Figure 3:**
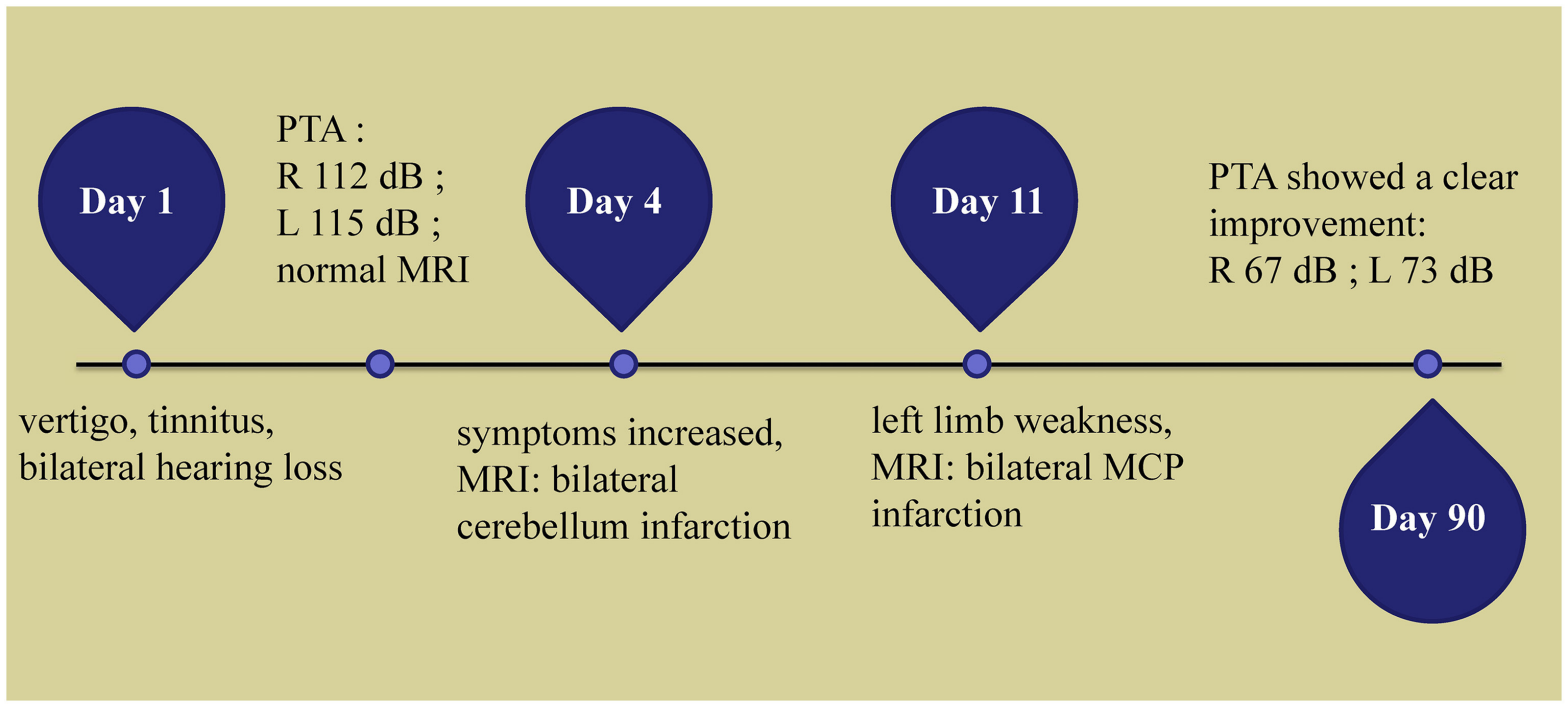
Time course of the disease. Diagram illustrating time course of disease in our patient. PTA, pure-tone average; R, right; L, left; MRI, magnetic resonance imaging; MCP, middle cerebellar peduncle.

The patient's symptoms improved after several days of antiplatelet, lipid-lowering, steroids and hyperbaric oxygen therapy. The patient was given oral prednisone at a dose of 60 mg for 4 consecutive days, followed by a reduction of 10 mg every 2 days. Hyperbaric oxygen therapy was administered once a day for 10 sessions. Three months later, Pure Tone Audiogram showed that SSNHL persisted; however, there was a clear improvement, with PTA average of 67 dB on the right and 73 dB on the left (Figure 1B). The speech audiometry showed 55 dB with 100% speech discrimination on the right and 60 dB with 100% speech discrimination on the left. His left-limb strength and coordination improved steadily over 3 months. The patient had no complaints at the 2-year follow-up visit to the stroke outpatient clinic. However, the patient continued to experience bilateral hearing loss, without any new neurological events. Unfortunately, we did not have access to his new PTA results at the 2-year follow-up. A time-course diagram of disease progression is shown in Figure 3.

## 3. Discussion

Bilateral SSNHL is rare, accounting for approximately 4.9% of all SSNHL (3), and cerebrovascular disease is more likely to be overlooked in this group of patients. When patients present with vertigo and sudden hearing loss without other neurological signs and symptoms, they are usually first seen in the ENT department. In a retrospective analysis of 103 case reports associated with bilateral SSNHL, the vascular condition was diagnosed as a pathophysiological factor in 17 patients (16.5%) (8). Vertigo and sudden hearing loss are the only signs of early AICA stroke (9), and AICA territory is most commonly associated with hearing disorders.

Bilateral sudden hearing loss with acute MCP infarction has been described in several reports, of which audiological examinations were performed in three cases (Table 1) (4–7). All patients were middle-aged to elderly, with a mean age of 57.3 years and with a male-to-female ratio of 3:1. Mid-total hearing impairment and vertigo were the most common symptoms, with facial palsy (*n* = 1), facial sensory disturbance (*n* = 1), ataxia (*n* = 3), and nystagmus (*n* = 4), also reported. All new AICA infarcts were seen from 0 to 7 days after the onset of symptoms, such as hearing loss. Two patients (patients 2, 4) had moderately severe to total hearing loss with normal BAEP, and our patient had normal electrocochleography, abnormal otoacoustic emissions indicating cochlear dysfunction and horizontal semicircular canal low-frequency dysfunction; one patient (patient 3) had moderate to moderately severe hearing loss with good speech discrimination. All these findings suggested that the hearing impairment was localized to the cochlea, and the central system function was intact. The patients' other neurological symptoms were mild and most of the hearing impairment had improved significantly (mild to severe hearing loss) at follow-up. Patients are detected early, without vertigo, with mild hearing loss and an upwards-sloping audiogram, which is considered to be associated with a good prognosis (10). Timely treatment of steroids and hyperbaric oxygen therapy can help hearing recovery (11). Previous studies have reported an improvement rate of 69.2% for the treatment of idiopathic SSNHL with hyperbaric oxygen in combination with intravenous steroids (11). Lee et al. in their summary of hearing loss with MCP infarction demonstrated that mild hearing loss with severely impaired speech discrimination and absence of auditory evoked responses are typical findings in central lesions such as stroke affecting the vestibulocochlear nucleus (12). Another patient (patient 1), with bilateral PTA ≥ 95 dB showed absence of otoacoustic emissions, no waveforms in BAEP, and bilateral MCP infarction involving the bilateral cochlear and inferior vestibular nucleus. The patient's hearing loss was considered to be central or mixed and her recovery was found to be poor at the 10-month follow-up. The audiogram showed no change in auditory threshold.

**Table 1:** Reviewing the literature on bilateral MCP with audiological examination.

**References**	**Bovo et al. (4)**	**Renard et al. (5)**	**Lee et al. (6)**	**Present study**
No.	Patient 1	Patient 2	Patient 3	Patient 4
Age	65	54	66	44
Gender	Female	Male	Male	Male
Risk factors	H, CA	H, HC, S	D	H
Vertigo	(+)	(+)	(+)	(+)
Ear symptoms	Binaural tinnitus and SSNHL	Bilateral SSNHL	Bilateral SSNHL, tinnitus in right ear	Binaural tinnitus and SSNHL
Nystagmus	Horizontal right-beating nystagmus	Gaze-evoked nystagmus	Spontaneous left-beating nystagmus	Gaze-evoked nystagmus
Other symptoms and signs	-	Ataxia	Diminished right-sided facial sensation; right peripheral facial palsy; ataxia	Ataxia
Grades of hearing loss (7)	Total hearing loss	Profound hearing loss	Moderate (L), moderately severe (R) hearing loss	Total hearing loss
Which ear	Both	Both	Both	Both
Degree (dB HL)	R, L ≥ 95	R 80; L > 90	R 55; L 45	R, L ≥ 95
Bithermal caloric test	-	-	right horizontal semicircular canal low-frequency dysfunction	Bilateral horizontal semicircular canal low-frequency dysfunction
BAEP	Absent at the maximum intensity	Normal	-	Normal
Localization of hearing loss	Central or mixed mechanism	Cochlea	Cochlea	Cochlea
Lesion location	Bilateral MCP	Bilateral MCP and the anterior inferior cerebellum	Bilateral MCP, right lateral pons	Bilateral occipital lobe and cerebellum, left thalamus and bilateral MCP
Vascular abnormality	MRA, brain angiograms: occlusion of BA	MRA, CTA: multifocal vertebrobasilar stenosis	MRA: moderately severe stenosis of distal RVA and the middle third of BA	CTA: occlusion of the bilateral VA V3, proximal V4 and bilateral AICA, stenosis at the distal LVA V4
Follow up	no improvement in the auditory threshold after 10 months	-	Vertigo, nausea and the level of hearing recovery in the left ear was partial recovery at discharge	the levels of hearing recovery in the both ears were slight improvement after 3 months

D, diabetes; H, hypertension; CA, cerebrovascular accident; HC, hypercholesteremia; S, smoke; -, not obtained; R, right; L, left; TC, total cholesterol; LDL-C, low-density lipoprotein cholesterol; TIA, transient ischemic attack.

All patients had one or more risk factors associated with systemic diseases, such as hypertension, diabetes, or hyperlipidemia. This is consistent with the literature, which reported that 38% of bilateral SSNHL were associated with such systemic diseases (13). Most angiographic findings were suggestive of vertebrobasilar artery stenosis and occlusion. In most cases, bilateral AICA emanate symmetrically from the BA. Almost all MCP is supplied by the AICA. Owing to severe stenosis or occlusion of the vertebrobasilar artery, when the lesion is in the vicinity of the inferior segment of the BA branching off the bilateral AICA, it tends to cause ischemia in the IAA supplying the inner ear. Damage to auditory hair cells and vestibular organs of the inner ear, which are extremely sensitive to ischemia and hypoxia, can cause SSNHL and vertigo. When the IAA is occluded at an early stage, it may only present with hearing loss or vertigo without the formation of intracerebral infarction. This situation should alert physicians. When the patient presents with hearing loss but no infarct foci on head MRI, and a three-step bedside oculomotor exam do not all suggest central disease, the patient should be closely monitored for changes in the condition and possible subsequent AICA infarction. Our patient's initial symptoms were vertigo, tinnitus and bilateral hearing loss, with no significant abnormalities on the head MRI. There was a change in condition on days 3 and 10, and the repeated head MRI suggested bilateral cerebellar and bilateral MCP infarcts, respectively. Therefore, we consider the hearing loss to be the prodromal symptom, with bilateral cerebellar and MCP infarcts occurring sequentially after the hearing loss at time intervals.

The purpose of this case report was to characterize the nature of deafness in the patient by performing detailed audiological and neurological investigations, such as the head MRI, pure tone audiometry, speech audiometry, acoustic immittancemetry, BEAP, and otoacoustic emissions to define the location of lesions associated with hearing loss. Our patient had persistent, bilateral, severe hearing loss on PTA, but limb strength and coordination gradually resolved over time, with no significant abnormalities in BEAP. At the same time, the symptoms of the facial and abducens nuclei and sensory tracts did not manifest during the course of the disease. We determined that bilateral hearing loss was affecting the cochlear rather than the retrocohlear sites such as the vestibulocochlear nerve, brainstem nucleus or other central pathways, as a result of ischemic infarction. Moreover, the retrocochlear acoustic nerve is believed to have an abundant collateral blood supply, which has a lower probability of injury (14). Meanwhile, the fact that patients are less sensitive to hearing loss than vestibular symptoms, has led to a lower incidence of auditory symptoms, as described in previous case reports of AICA infarction (15). Audiological examinations should not be neglected in patients with MCP infarction, as it is important for the localization of the disease.

Moreover, our patient had persistent bilateral deafness 10 days before the diagnosis of bilateral MCP infarction, which was a much longer interval than previous bilateral MCP infarction cases. In a study of 16 patients with unilateral AICA infarcts, isolated recurrent vertigo, fluctuating hearing loss, and tinnitus, as the first symptoms, occurred in 31% (*n* = 5) of patients within 1–10 days before the onset of the brainstem and cerebellar symptoms (15). In 82 cases of AICA infarcts, MRA of patients with and without prodromal symptoms of the auditory vestibular system showed that stenosis of the basilar artery near the beginning of AICA was more common in the former (62 vs. 13%) (16). Bilateral AICA occlusion in our patient was considered to be related to severe VA stenosis with thrombosis obstructing the orifices of both AICA, which is consistent with the mechanism reported by Ogawa et al. (17). Zhang et al. confirmed the value of basi-parallel anatomic scanning magnetic resonance imaging (BPARS-MRI) in clarifying the diagnosis of the vascular etiology of AICA infarcts, which can reveal an accurate outer contour of the vascular wall (18).

We describe the case of a patient with bilateral symmetric MCP infarction who presented with sudden bilateral deafness as the initial symptom. Vertebrobasilar diseases should be routinely considered in middle-aged and elderly patients with vascular risk factors and bilateral hearing loss. Diagnosis can easily be delayed when patients have only symptoms such as hearing loss and vertigo, with few typical stroke symptoms. Brain MRI, brain MRA, BAEP, otoacoustic emissions, and Pure Tone Audiogram help to localize and qualify the diagnosis. We emphasized the cochlear, not the retrocochlear, as the localization of bilateral deafness in this patient with bilateral MCP infarction. Early detection of hearing loss and intervention can help patients recover. Bilateral hearing loss localized to the periphery usually improves better after treatment and has a better prognosis.

## Data availability statement

The original contributions presented in the study are included in the article/supplementary material, further inquiries can be directed to the corresponding author.

## Ethics statement

Ethical review and approval was not required for the study on human participants in accordance with the local legislation and institutional requirements. The patients/participants provided their written informed consent to participate in this study. Written informed consent was obtained from the individual(s) for the publication of any potentially identifiable images or data included in this article.

## Author contributions

Material preparation was performed by RL. Data analysis was performed by ZY and LX. The first draft of the manuscript was written by ZY. WY contributed to the study conception and approved the final manuscript. All authors commented on previous versions of the manuscript. All authors contributed to the article and approved the submitted version.
